# Role of Habenula in Social and Reproductive Behaviors in Fish: Comparison With Mammals

**DOI:** 10.3389/fnbeh.2021.818782

**Published:** 2022-02-10

**Authors:** Satoshi Ogawa, Ishwar S. Parhar

**Affiliations:** Brain Research Institute, Jeffrey Cheah School of Medicine and Health Sciences, Monash University Malaysia, Subang Jaya, Malaysia

**Keywords:** neuropeptides, social behavior network, teleost, estrogens, social decision-making network

## Abstract

Social behaviors such as mating, parenting, fighting, and avoiding are essential functions as a communication tool in social animals, and are critical for the survival of individuals and species. Social behaviors are controlled by a complex circuitry that comprises several key social brain regions, which is called the social behavior network (SBN). The SBN further integrates social information with external and internal factors to select appropriate behavioral responses to social circumstances, called social decision-making. The social decision-making network (SDMN) and SBN are structurally, neurochemically and functionally conserved in vertebrates. The social decision-making process is also closely influenced by emotional assessment. The habenula has recently been recognized as a crucial center for emotion-associated adaptation behaviors. Here we review the potential role of the habenula in social function with a special emphasis on fish studies. Further, based on evolutional, molecular, morphological, and behavioral perspectives, we discuss the crucial role of the habenula in the vertebrate SDMN.

## Introduction

Social and reproductive behaviors are essential biological processes for the survival of individuals or species in vertebrates and invertebrate species. In vertebrates, the neural foundation of the social and reproductive behaviors is controlled by the brain circuitry that is called the social behavior (or brain) network (SBN) (Newman, 1999). The SBN is evolutionarily, morphologically, biochemically and functionally conserved throughout vertebrate species (Goodson, 2005; O’Connell and Hofmann, 2011b,^2012^; Goodson and Kingsbury, 2013; Ogawa et al., 2021). The SBN primarily consists of cellular components sensitive to sex steroids and neuropeptide hormones, which regulate innate social behaviors such as sexual behavior, aggression, and parental care (Newman, 1999; Goodson, 2005). On the other hand, despite the conserved structure of the SBN, phenotypes and functions of social and reproductive behaviors are far more diverse and complex depending on species, genera, sex, and sometimes among individuals. In humans, social functions are highly complex, which have also been implicated in mental and psychiatric disorders associated with social deficits (Heinrichs et al., 2009). In particular, the SBN can drive the behavioral output of social functions, while it does not seem to be enough to drive the evaluation of social contexts. Hence, the cross-talk between the SBN and the mesolimbic reward system, which mainly consists of the dopaminergic system, has emerged as a more extensive important network for the social function, called the social decision-making network (SDMN) (O’Connell and Hofmann, 2011a,b, 2012).

Wei et al. (2021) systematically categorized the process of the goal-directed innate social behavior in vertebrates based on four stages: (I) *detection phase*, in which an individual identifies the presence and location of a distant social target *via* unique sensory cues emitted by the target; (II) *approach phase*, which is to reduce the distance between an individual and a distal social stimulus for a prerequisite of the remaining phases; (III) *investigation* (social investigation) phase is for further close exploration of the social stimulus, aiming to gather information about the conspecific; and (IV) *consummatory action phase* is to accomplish the goal of social behavior, which differs depending on types of behaviors. These four phases are selectively regulated by the different nodes of the SDMN and their stimulatory or inhibitory connectivities (Wei et al., 2021). Depending on various factors including the type of external and internal inputs, social experiences, hormonal action and molecular/neuronal modifications of the node and pathway, different types of social behavioral outputs can be generated by the SDMN (Wei et al., 2021). Even within the same individual or sex, the same node could also play multiple roles dependent on hormonal, environmental and social experiences. For example, during the consummatory action phase, the ventrolateral part of the ventromedial hypothalamus (VMHvl) is essential for aggression and female sexual behaviors (Yang et al., 2013; Veening et al., 2014), and the medial preoptic area (MPOA) is necessary for parental behaviors and male sexual behavior (Hull and Dominguez, 2007).

However, how the SDMN could generate such a large diverse behavioral phenotypes among individuals, sex, and species remains unknown. A systematic comparative analysis has enabled us to identify conserved molecular and neuronal pathways that regulate a particular type of behavior in diverse species or other sublineages (Toth and Robinson, 2007). Teleost fish represent the largest and most diverse group among vertebrates, consisting of nearly 30,000 species (Ravi and Venkatesh, 2018). Teleosts exhibit various social and reproductive behaviors unique to species or genera (Brown et al., 2011; Ogawa et al., 2021). Hence, the teleost is an ideal model to identify the conserved neuronal and molecular mechanism controlling the social and reproductive behaviors and the regulatory mechanism for generating diverse behavioral phenotypes.

Recently, the habenula, an evolutionarily conserved epithalamic structure, has been implicated in social and reproductive behaviors in rodents and teleosts (Ogawa et al., 2021). The habenula encodes both the social context of rewarding and aversive aspects of external stimuli, thus driving motivated behaviors and decision-making in vertebrates (Boulos et al., 2017; Fore et al., 2018). In rats, the habenula is implicated in the positive aspect (rewarding effect) of social behaviors such as the onset of maternal behaviors and social play (Corodimas et al., 1992, 1993; Matthews-Felton et al., 1995; Felton et al., 1998; van Kerkhof et al., 2013). On the other hand, the habenula is also involved in the negative (stressful effect) aspect of social functions such as social isolation (de Jong et al., 2010), anxiety and fear (Yamaguchi et al., 2013), and aggression (Flanigan et al., 2017). Interestingly, the habenula also plays an emerging role in reward and stressful social events (e.g., rewarding context of aggression) (Flanigan et al., 2020). In zebrafish (*Danio rerio*), the habenula is involved in aversive social responses such as anxiety (Mathuru and Jesuthasan, 2013), fear (Agetsuma et al., 2010), and aggression (Chou et al., 2016). The habenula pathway is structurally and neurochemically conserved in vertebrates (Aizawa et al., 2012; Beretta et al., 2012; deCarvalho et al., 2014), and hence, it is reasonable to consider the possible connection between the habenula and the SDMN (Ike et al., 2020; Ogawa et al., 2021). In this review article, we summarize the potential role of the habenula in social function with particular emphasis on fish studies. Further, based on evolutional, molecular, morphological and behavioral perspectives, we discuss the crucial role of the habenula in the vertebrate SDMN.

## Social Decision-Making Network in Vertebrates

### Social Decision-Making Network in Mammals

**FIGURE 1:**
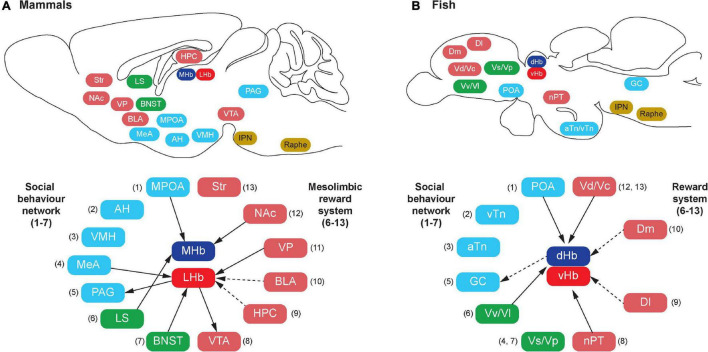
Schematic illustration of habenula connectivity with the social decision-making network (SDMN) in vertebrates. In mammals (A), the SDMN comprises the social behavior network (SBN) (1–7) and the mesolimbic reward structures (6–13), but some structures are shared by the SBN and the mesolimbic reward structures (6 and 7). In teleosts (B), each brain region that has been suggested to be homologous to the respective components of the mammalian SDMN is indicated by the corresponding number (shown in parentheses). In mammals, the habenula consists of the medial (MHb) and lateral (LHb) subnuclei, which receive direct (solid lines) or indirect (dotted lines) projection from the component of SDMN. In teleosts, the habenula consists of the dorsal (dHb) and ventral (vHb) subnuclei, which are homologous to mammalian MHb and LHb, respectively. Similar to mammals, there is connectivity between the habenula and teleostean SDMN. However, the detailed subnuclei-specific connectivity has not been precisely characterized in fish brain. The abbreviation of brain regions refers to Table 1.

In mammals, the SBN consists of the preoptic area (POA), lateral septum (LS), anterior hypothalamus (AH), ventromedial hypothalamus (VMH), extended medial amygdala [containing the medial amygdala (MeA) and medial bed nucleus of the stria terminalis (mBNST or BSTm)], and periaqueductal gray (PAG) (Newman, 1999; Figure 1A and Table 1). Each of the brain regions is sensitive to sex steroid hormones and has been implicated in the control of multiple forms of social behavior including aggression, sexual behavior, and various forms of social communication such as social recognition, affiliation, bonding, parental behavior and social stress responses (Goodson, 2005). In mammals, each node for the SBN is interconnected and the SBN also emerges with intrinsic and extrinsic systems (Rogers-Carter and Christianson, 2019), which allows performing complex social cognition and its associated behaviors (Rilling and Sanfey, 2011; Mars et al., 2012). For instance, there are functional connectivities between the SBN and the reward center (O’Connell and Hofmann, 2011b), the corticostriatal pathway (Felix-Ortiz and Tye, 2014; Ko, 2017), the nigrostriatal pathway, the mesocortical pathway (Skuse and Gallagher, 2009) and the default mode network (Mars et al., 2012). The connection between the SBN and the mesolimbic reward structures composes the SDMN (O’Connell and Hofmann, 2012; Figure 1A and Table 1), which modulates behavioral reactions in a social situation reflect the convergence of social information, external stimuli and physiological state to produce appropriate responses (Rogers-Carter and Christianson, 2019). The expression profiles of several genes involved in the SDMN in 88 species across five vertebrate lineages confirmed a high level of conservation of the SDMN in vertebrates (O’Connell and Hofmann, 2012).

**TABLE 1:**
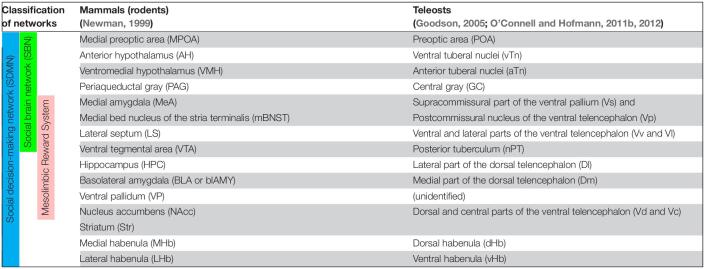
Summary of proposed homologies of between brain nuclei/regions in social decision-making network of mammals (rodents) and teleosts.

### Social Decision-Making Network in Fish

In teleosts, several brain regions corresponding to mammalian SDMN have been characterized based on comparative neuroanatomy (Braford, 1995; Wullimann and Rink, 2002), pharmacological, neurodevelopmental, neurochemical, neurophysiological and genetic approaches (Chandroo et al., 2004; Maximino et al., 2013; do Carmo Silva et al., 2018) as summarized in Table 1. Among the component of the SDMN, only limited nodes such as the POA and the PAG (=griseum centrale/central gray, GC) are structurally and biochemically well conserved in the fish brain (Goodson, 2005; O’Connell and Hofmann, 2011b; Ogawa et al., 2021; Figure 1B). On the other hand, some nodes in the SBN such as the AH, VMH, MeA, BNST and LS, and major nodes of SDMN [e.g., hippocampus, ventral tegmental area (VTA), and nucleus accumbens core (NAc)] in tetrapods have not been structurally well defined in the fish brain (Goodson and Kingsbury, 2013). However, based on molecular, developmental and functional characterizations, the teleostean homolog of many tetrapod-specific brain regions have been proposed (Northcutt, 2011; Goodson and Kingsbury, 2013; Figure 1 and Table 1). In the hypothalamus, the anterior and ventral tuberal nuclei (aTn and vTn) are suggested as the mammalian VMH and AH homologs, respectively (O’Connell and Hofmann, 2011b). Developmental and gene expression studies have pointed to the supracommissural part of the ventral pallium (Vs) as the putative homolog of the extended medial amygdala (Ganz et al., 2012; O’Connell and Hofmann, 2012). However, the Vs is also considered as a part of the postcommissural nucleus of the ventral telencephalon (Vp) and the Vs/Vp has been suggested to be a homolog of the entire amygdala, which includes the central amygdala, MeA and BNST (Reiner and Northcutt, 1992; Northcutt, 1995). On the other hand, the ventral and lateral parts of the ventral telencephalon (Vv and Vl) have been suggested as putative homologs of the LS based on biochemical and functional evidence (O’Connell et al., 2012; Goodson and Kingsbury, 2013).

The presence of teleostean homologs of the mammalian mesolimbic reward structures including the hippocampus, VTA, ventral pallidum (VP), striatum, NAc, and basolateral amygdala (BLA) are debatable because of their lack of structurally homologous regions in fish brain. However, the number of accumulated functional and neurochemical evidence suggest their presence in the fish brain (O’Connell and Hofmann, 2011b). The lateral part of the dorsal telencephalon (Dl) is considered to be the putative homolog of the tetrapod hippocampus based on brain circuitry and functional studies (O’Connell and Hofmann, 2011b). In goldfish (*Carassius auratus*), lesion of the Dl area results in the impairment of spatial-temporal and emotional learning (Portavella et al., 2002). Similarly, the lesions of specific locations in the telencephalic pallia in goldfish suggest the presence of two different memory systems in fish: the medial telencephalic pallia is involved in an emotional memory system, while and the lateral telencephalic pallia are involved in a spatial, relational, or temporal memory system (Portavella et al., 2004).

The teleostean homolog of the VTA has long been debatable as the midbrain (VTA- substantia nigra) dopaminergic neurons are absent in the fish brains. However, the dopaminergic neurons in the posterior tuberculum (nPT, also known as periventricular posterior tuberculum) have been suggested as a possible homolog of mammalian midbrain dopaminergic population because their projections ascend to the striatal-like structure, similar to mammals (Rink and Wullimann, 2001, 2002; Matsui, 2017). In addition, in the brain of the African cichlid fish, *Astatotilapia burtoni*, three regulatory genes (*etv5*, *nr4a2*, and pitx3) important for maturation and maintenance of midbrain dopaminergic cells in mammals are present in several dopaminergic cell groups located in the POA, rostral periventricular pretectal nucleus, posterior tuberal nucleus, and the nPT (O’Connell et al., 2013). Hence, although the location of dopaminergic cell populations is extremely variable across vertebrate species (O’Connell and Hofmann, 2012), dopaminergic cell groups are evolutionarily and functionally conserved across vertebrates.

In goldfish and zebrafish, the medial part of the dorsal telencephalon (Dm), a subregion of the Vp has been postulated to be essential for emotional learning and related processes (i.e., avoidance learning), suggesting that the Dm could be functionally homologous to the mammalian BLA (Portavella et al., 2004; Portavella and Vargas, 2005; Maximino et al., 2013; Lal et al., 2018). Based on neurochemical evidence, the dorsal (Vd) and central (Vc) parts of the ventral telencephalon have been suggested as putative striatal-like structure in fish (Wullimann and Mueller, 2004). Collectively, key nodes of the SDMN are evolutionarily conserved in tetrapods and in the fish brain (O’Connell and Hofmann, 2011b; Rodriguez-Santiago et al., 2021).

## Possible Connection Between the Habenula and the Social Decision-Making Network

### Structure of the Habenula

**FIGURE 2:**
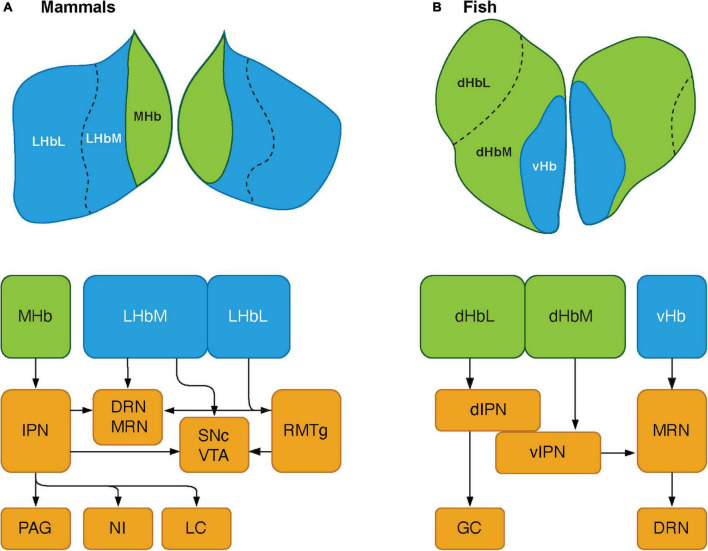
Schematics showing the habenular pathways in mammals and teleosts. (A) The habenula consists of two major subnuclei, medial habenula (MHb) and lateral habenula (LHb) in mammals. LHb can be further divided into medial (LHbM) and lateral (LHbL) parts. MHb primarily projects to serotonergic center: the dorsal and median raphe nuclei (DRN and MRN), and dopaminergic center: substantia nigra pars compacta (SNc) and ventral tegmental area (VTA) *via* the interpeduncular nucleus (IPN). The MHb also sends projections to other brain regions such as the periaqueductal gray (PAG), nucleus incertus (NI), and locus coeruleus (LC). LHb projects serotonergic raphe nuclei and midbrain dopaminergic center. However, LHbL act on serotonergic and dopaminergic centers *via* the rostromedial tegmental nucleus (RMTg, which is also known as tail of the VTA). (B) In teleosts, the habenula structure consists of two subnuclei: dorsal habenula (dHb) and ventral habenula (vHb), which are structurally and biochemically homologous to mammalian MHb and LHb, respectively. dHb and their pathways are structurally asymmetric, hence they can be considered subnuclei: lateral (left) and medial (right) dHb (dHbL and dHbM). dHbL and dHbM project to the different regions of IPNs: dorsal (dIPN) and ventral (vIPN) part of the IPN, respectively. dIPN further innervates to the griseum centrale (GC, a homolog of the mammalian PAG), while vIPN is connected to the MRN. vHb sends projection to the ventro-anterior corner of the MRN, which may further extend toward the serotonergic DRN. Modified from Ogawa et al. (2021).

Although the key nodes of the SDMN are biochemically and functionally conserved in the vertebrate brains, they have functional connectivity with several other brain regions that reside outside the SDMN. The habenula is part of the epithalamic structure, a dorsal posterior segment of the diencephalon (Dicke and Roth, 2007). The habenula is involved in several functions, including regulating midbrain monoaminergic systems (dopamine and serotonin) and integrating cognitive with emotional and sensory processing (Boulos et al., 2017). In mammals, the habenula consists of two major subnuclei, lateral (LHb) and medial (MHb), which have different circuitry (afferents and efferents), chemical components and selective functions (Hikosaka, 2010; Figure 2A). The structure of the habenula is highly conserved in non-mammalian vertebrates. In fish and amphibians, the habenula is subdivided into dorsal (dHb) and ventral habenula (vHb) subnuclei, based on their cytoarchitectural and molecular characteristics (Figure 2B; Pandey et al., 2018). In non-mammalian vertebrates, the dHb is structurally asymmetric (either left-directed or right-directed) with different volumes of nuclei and their respective efferent neural circuitry, while the vHb is symmetric (Concha and Wilson, 2001). Genetic mechanisms play a major role in generating the asymmetric habenular structure. The left-right differences in the number of neurons are controlled by the regulation of developmental processes such as proliferation, differentiation, migration, and cell death (Aizawa, 2013). In zebrafish, the dHb is a left-directed asymmetric structure, which can be further subdivided into the lateral (dHbL) and medial (dHbM) subnuclei (Aizawa et al., 2005). Based on morphological, molecular and biochemical features, the dHb and vHb have been characterized as homologous regions of the mammalian MHb and LHb, respectively (Aizawa et al., 2005; Amo et al., 2010; Pandey et al., 2018). Several selective markers for the habenula have been identified, which exhibit similar expression patterns in mammals and teleosts (Aizawa and Zhu, 2019). GPR151, an orphan G protein-coupled receptor (GPCR), is a selective marker for the MHb in rodents (Broms et al., 2015), and is expressed in the dHb in the zebrafish (Broms et al., 2015). Protocadherin 10a (Pcdh10), a selective marker for the LHb in rats is expressed in the vHb in the zebrafish (Amo et al., 2010; Aizawa et al., 2012). On the other hand, Brn3a (POU domain, class 4, transcription factor 1), a selective marker for the MHb in rodents (Quina et al., 2009) is expressed only in the dHbL in the zebrafish (Aizawa et al., 2005). Similarly, GPR139, another orphan GPCR rich in the MHb in rodents (Süsens et al., 2006; Liu et al., 2015), is expressed only in the vHb in the zebrafish (Roy et al., 2021). *kiss1* is a gene encoding neuropeptide, kisspeptin, which is predominantly expressed in the vHb in some teleosts (Beretta et al., 2013; Ogawa and Parhar, 2013; Pandey et al., 2018), is absent in the habenula of mammalian species. Hence, the cytoarchitectural and the molecular make-up of the habenula is relatively conserved with minor variances among vertebrate species.

### Neural Circuitry of the Habenula and Their Connection With the Social Decision-Making Network

Similar to the habenular structures, the neural circuitry of the habenula in teleosts is also comparable to those in mammals with minor variances (Yañez and Anadón, 1996; Hendricks and Jesuthasan, 2007; Hikosaka et al., 2008; Miyasaka et al., 2009; Gayoso et al., 2011; Amo et al., 2014; Namboodiri et al., 2016; Turner et al., 2016; Figure 2). In vertebrates, the habenula has a structural connection with the SDMN (Figure 1). The LHb has been suggested as one of the core cortical components of Brothers’ (Brothers, 1990) and Dunbar’s (Dunbar, 2009) Cognitive Social Brain (Ike et al., 2020). In mammals, the MHb receives projections from several nodes of the SDMN including the POA, LS, and NAc (Figure 1A), and the LHb also receives inputs from the SDMN including the MeA, VP and BNST (Boulos et al., 2017; Figure 1A). In addition, the LHb also act on the SDMN nodes including the VTA and PAG *via* the rostromedial tegmental nucleus (RMTg) (Hikosaka et al., 2008; Graziane et al., 2018). Although there are no direct connections between the habenula and the hippocampus, electrical or iontophoretic activation of LHb modulates hippocampal pyramidal cell activity, which suggests functional coupling of LHb-hippocampus *via* indirect connection (Ferraro et al., 1997; Geisler and Trimble, 2008; Goutagny et al., 2013).

Although the selective origin from or terminal projection sites within the habenula nuclei remains uncharacterized in fish, neural tracer studies in fish have revealed afferent connectivity of the habenula with some nodes of the fish SDMN such as the Vv (the teleostean homolog of the LS), Vd (the homolog of mammalian striatum), and POA (Villani et al., 1996; Yañez and Anadón, 1996; Turner et al., 2016; Figure 1B). A recent functional study demonstrated that microstimulation of the Dl and Dm (the homolog of mammalian hippocampus and BLA) elicit significant activation in the habenula neurons in juvenile zebrafish (Bartoszek et al., 2021). Further, ongoing neural activities in the dHb are significantly correlated with the neural activities in the Dm and olfactory bulb, while vHb activity correlates more with the activity in the Dl rather than those in the Dm (Bartoszek et al., 2021). Although no direct anatomical projections from the Dl or the Dm have been identified to innervate the habenula in fish, the primary afferents of the vHb originates from the ventral entopeduncular nucleus (vENT = teleostean basal ganglia) in zebrafish (Amo et al., 2014; Turner et al., 2016). Further, the vENT receives direct innervation from the Dm and Dl in fish (Echteler and Saidel, 1981; Northcutt, 2006; Lal et al., 2018). Hence, there might be an indirect connection between the habenula and the Dm/Dl regions in fish (Figure 1B).

The habenula nuclei project to different targets in the brain of teleosts, similar to mammals. In the zebrafish, the dHbL and dHbM terminate in the dorsal and ventral part of the IPN (dIPN and vIPN), respectively (Aizawa et al., 2005; Villalón et al., 2012; Figure 2B). The dIPN neurons further project to the dorsal part of the superior raphe, which terminates at the GC (the homolog of mammalian PAG) (Yañez and Anadón, 1996; Agetsuma et al., 2010), while the vIPN is reciprocally connected with the median raphe (MR) (Agetsuma et al., 2010). In contrast, the vHb directly projects to the ventro-anterior region of the MR (vaMR) (Amo et al., 2010; Nathan et al., 2015). In mammals, there is connectivity between the habenula and the mesolimbic (VTA-SNc) dopaminergic system (Christoph et al., 1986; Brinschwitz et al., 2010). However, the habenula receives afferent projection from the nPT (the homolog of mammalian VTA) in fish (Yañez and Anadón, 1996; Turner et al., 2016). These results suggest that the innervations from the SDMN to the habenula in fish is structurally and functionally comparable to those in mammals (Figure 1).

In addition to the connection with the SDMN, the habenula also receives direct inputs from the mitral cells of the olfactory bulbs (OB) in the zebrafish (Miyasaka et al., 2009, 2014; deCarvalho et al., 2013). The olfactory inputs are important cues for the animal’s social behaviors and the MeA receives direct inputs from the OB in mammals (Keshavarzi et al., 2015). Further, the activities of the habenula, in particular, dHb neurons are triggered by a variety of sensory cues including olfactory (Jetti et al., 2014; Krishnan et al., 2014; Chen et al., 2019), visual (Dreosti et al., 2014; Cherng et al., 2020) and non-visual cues (Lin and Jesuthasan, 2017; Eilertsen et al., 2021). The OB-dHb pathway is also sensitive to the chemical components of alarm pheromone or substances, a mixture of substances called Schreckstoff in German (von Frisch, 1941) that triggers fear-like avoidance behavior in zebrafish (Jesuthasan and Mathuru, 2008; Mathuru et al., 2012; Jetti et al., 2014; Choi et al., 2021). However, the role of the OB-habenula circuitry in the modulation of social behaviors associated with olfactory signaling such as sexual behavior and social avoidance remains unknown.

## Regulators of Reproductive and Social Behaviors on the Habenula

### Gonadal Steroid Sensitivities of the Habenula

Gonadal (sex) hormones such as estrogens, androgen and progesterone are key regulators of reproductive and social behavior in vertebrates (Adkins-Regan, 2009; Jennings and de Lecea, 2020). The SDMN functions are sensitive to gonadal steroids, which are modulated *via* steroid hormone receptors. In addition, the habenula is also sensitive to gonadal hormones (Pfaff and Keiner, 1973; Yokosuka et al., 1997; Shughrue et al., 1998; Wagner et al., 1998; Pérez et al., 2003; Shima et al., 2003). In rodents, there are two estrogen receptor (ER) types (ERα and ERβ), which are present in the LHb neurons (Pfaff and Keiner, 1973; Yokosuka et al., 1997; Shughrue et al., 1998; Wagner et al., 1998; Pérez et al., 2003; Shima et al., 2003). In the brain of the postnatal mouse (P9), ERβ-immunoreactive cells are detected in the MHb (Sugiyama et al., 2009). In female rats, ERβ expression in the habenula is upregulated under low estrogen levels, but they are significantly reduced when 17β-estradiol is supplemented (Shima et al., 2003). Expression of receptors for androgen (AR) and progesterone (PR) in the habenula has also been demonstrated in some mammalian species. In the rat brain, AR and PR mRNA is expressed in the habenula (Simerly et al., 1990; Hagihara et al., 1992; Kato et al., 1994). In addition, there is functional evidence for the sensitivity of habenula to androgens such as the androgen-binding activity in the MHb of the golden hamster (Doherty and Sherdan, 1981) and Fos induction in the LHb followed by testosterone administration in male Syrian hamster (DiMeo and Wood, 2006).

In some fish, ERs and AR are also present in the habenula, but only in limited species. In goldfish, ER types (*era1* and *erb1*) genes are expressed in the vHb (Wang et al., 2013). In *A. burtoni*, ERα, ERβ, ARα, and ARβ (mRNA and immunoreactivity) are expressed in the habenula (Munchrath and Hofmann, 2010). However, in medaka and the midshipman fish (*Porichthys notatus*), expression of ERα and AR mRNA is absent in the habenula (Forlano et al., 2005, 2010; Mitani et al., 2010). In zebrafish, treatment with a selective agonist for ERβ (WAY-200070) significantly increases *c-fos* expression in the habenula (Chen et al., 2015). Hence, the habenula could possibly be involved in sex steroid-dependent modulation of reproductive and sexual behaviors in fish.

### Hypothalamic Neuropeptidergic Action on Habenula

The habenula also receives inputs from the diencephalic regions including the POA, which also play important roles in the neuroendocrine regulation of reproduction including sexual behavior in vertebrates. The POA–hypothalamic region contains several cell groups expressing key neuroendocrine regulators (neuropeptides) such as gonadotropin-releasing hormone (GnRH), kisspeptin, vasopressin (AVP) and oxytocin which are involved in the regulation of sexual behaviors in vertebrates (Insel and Young, 2000; Zohar et al., 2010; Gonçalves and Oliveira, 2011). In mammals and fish, the habenula receives fiber projections of neuropeptidergic neurons and expresses neuropeptides receptors, suggesting a possible role of neuropeptidergic action on the habenula in vertebrate sexual and social behaviors.

#### Gonadotropin-Releasing Hormone

Gonadotropin-releasing hormone is a hypothalamic neuropeptide, which is primarily involved in the control of the release of gonadotropin (luteinizing hormone, LH and follicle-stimulating hormone) from the anterior pituitary. In vertebrates, there are multiple GnRH paralogs and multiple receptor (GnRHR) types (Parhar, 2002; Okubo and Nagahama, 2008). In mammals, there are two GnRH paralogs (GnRH1 and GnRH2), while non-mammalian vertebrates possess two or three GnRH paralogs (GnRH1, GnRH2, and GnRH3) in the brain (Parhar, 2002; Oka, 2018). Each GnRH paralog is localized in different brain regions and exhibit different roles. The GnRH1 neurons in the preoptic area are hypophysiotropic in nature and function in the neuroendocrine control of reproduction, while the non-hypophysiotropic GnRH2/GnRH3 neurons play neuromodulatory roles in metabolism and social behavior (Ogawa et al., 2022).

In mammals, GnRH neurons are sensitive to estrogens and their terminals act on the median eminence to modulate pulsatile and surge release of LH *via* estrogen feedback mechanism. In addition, GnRH fibers and GnRHRs are widely distributed in many brain regions, suggesting their multiple functions. GnRH1 and GnRH2 have also been implicated in the modulation of female sexual behaviors in several species, such as musk shrews (Temple et al., 2003), rodents (Sakuma and Pfaff, 1980, 1983; Kauffman and Rissman, 2004), and primates (Kendrick and Dixson, 1985; Barnett et al., 2006). In some tetrapods, GnRH1 fibers are also present in the habenula (Merchenthaler et al., 1984; Muske et al., 1994; Rastogi et al., 1998). In rats, moderate to strong expression of GnRHR1 mRNA has been detected in the MHb (Jennes and Conn, 1994; Jennes and Woolums, 1994). On the other hand, in the transgenic mice, GnRHR1 promoter-driven yellow fluorescence protein expression is present in the LHb (Wen et al., 2011). Similarly, in musk shrews, GnRHR2 receptor is distributed in several brain regions including the POA, cingulate cortex, arcuate nucleus, infundibular stalk and habenula (Temple et al., 2003). Interestingly, the majority of GnRH2 fibers terminate in the MHb in musk shrews (Rissman et al., 1995). In marmoset, the promoting effect of GnRH2 on sexual behavior is not blocked with Antide, a potent antagonist for GnRHR1 (Barnett et al., 2006). Hence, GnRHR2 signaling in the habenula could be primarily involved in promoting the effect of GnRH1 or GnRH2 on female sexual behaviors.

The presence of GnRH fibers or GnRHR expression in the habenula has also been demonstrated in several teleosts. In zebrafish, goldfish and salmon (*Oncorhynchus masou*), GnRH2 and GnRH3 fibers are present in the habenula (Amano et al., 1991; Kim et al., 1995; Song et al., 2014; Xia et al., 2014). In *A. burtoni*, GnRHR2 mRNA is expressed in the habenula (Chen and Fernald, 2006), while in the sea lamprey (*Petromyzon marinus*), GnRHR1 and GnRHR2 are expressed in the habenula (Hall et al., 2013). However, it remains unknown whether GnRH fibers terminate and exhibit modulatory roles in the habenula in fish because GnRH fibers and GnRH receptor are also present in the pineal gland that receives direct afferent projections from the habenula (Park et al., 1995; Sakharkar et al., 2005; Servili et al., 2010; Schang et al., 2013). Unfortunately, no physiological and behavioral study has demonstrated the role of GnRH-GnRHR signaling in the habenula and its potential involvement in the modulation of sexual behavior in fish.

#### Kisspeptin

Kisspeptin is a relatively newly identified neuropeptide, originally identified as a gene product of metastasis suppressor gene, *KISS1* in human melanoma cells (Lee et al., 1996; Kotani et al., 2001). In 2003, two independent studies reported the role of kisspeptin and its cognate receptor, GPR54 (Kiss1R or Kissr) in reproductive function because of their role in GnRH-LH secretion during the pubertal onset in mammals (de Roux et al., 2003; Seminara et al., 2003). Gene synteny analyses revealed that the presence of four ancestral *Kiss* genes (*Kiss1*, *Kiss2*, *Kiss3*, and *Kiss4*) and respective *Kissr* orthologous genes (*Kissr-1*, *-2*, *-3*, and *-4*) in the vertebrate genome (Lee et al., 2009; Pasquier et al., 2012). *Kiss1* and *Kissr-1* genes are present in mammals, while the remaining paralogous genes might have been lost during evolution (Pasquier et al., 2012). Most teleosts possess one or two (*Kiss2* and/or *Kiss1*) *Kiss* paralogs together with multiple Kissr (Kissr2 and/or Kissr1) paralogs [also designated as Kissr2 and/or Kissr3, according to the nomenclatures by Pasquier et al. (2012)].

In rodents, Kiss neurons are localized in the hypothalamus with two distinct neuronal populations: one in the anteroventral periventricular nucleus [AVPV, a part of the rostral periventricular area of the third ventricle (RP3V)] and the other in the arcuate nucleus (ARC) (Lehman et al., 2010). In teleosts, expression patterns in the brain vary depending on Kiss paralog types and species (Ogawa and Parhar, 2013). Kiss2 is expressed in the preoptic-hypothalamic region in teleosts, which is conserved among teleost species (Ogawa and Parhar, 2013). On the other hand, Kiss1 is expressed in the POA and/or in the vHb (Ogawa and Parhar, 2013). Furthermore, in the brain of zebrafish, Kissr2 (the receptor exhibits higher affinity to Kiss2) gene is widely distributed, while Kissr1 (the receptor for Kiss1) is coexpressed with Kiss1 in the vHb (Kitahashi et al., 2009; Ogawa et al., 2012). In zebrafish, Kiss1 neurons in the vHb project their axons to the IPN *via* the fasciculus retroflexus, which terminate in the vaMR (Nathan et al., 2015). In some fish species that possess only Kiss2, Kissr1 or Kissr2 is also expressed in the habenula (Zmora et al., 2012; Ohga et al., 2017; Macedo-Garzón et al., 2021). In rodents, Kissr but not Kiss1 is expressed in the habenula (Lee et al., 1999; Herbison et al., 2010; Simonneaux et al., 2013; Wagner et al., 2016). Further, rabies viral tracer in mice revealed monosynaptic inputs from the IPN to caudal ARC Kiss1 neurons (Yeo et al., 2019). These suggest that Kiss1 derived from the ARC-Kiss1 neurons could act on the IPN/MR rather than the habenula in mice. The potential role of Kiss within the habenula pathway is not fully understood. However, in zebrafish, habenula Kiss1 neurons have been associated with the modulation of serotonin neurons, fear-like responses and aversive memory and learning (Ogawa et al., 2012, 2014; Lupton et al., 2017; Sivalingam et al., 2020) (see section “Fear- and Anxiety- Like Behaviors” for more details).

#### Neuropeptide Y

Neuropeptide Y (NPY) is one of the most evolutionarily conserved neuropeptides, which primarily regulates energy homeostasis (Mercer et al., 2011). In addition, NPY is also involved in various physiological and neuronal processes including reproductive and social behaviors (Shende and Desai, 2020). NPY activates its cognate GPCR, Y receptors (Y_1_-Y_6_) in different brain regions and cellular locations (Shende and Desai, 2020). In mice, Y1 and Y2 receptors, which are post-synaptic and pre-synaptic, respectively (Shende and Desai, 2020), are expressed in the LHb (Kopp et al., 2002; Stanić et al., 2006). In rats, exposure to NPY decreases the spontaneous firing rate of the LHb, leading to hypoactivation of the LHb, which is modulated by reduction of GABAergic transmission *via* the Y1 pathway (Cheon et al., 2019, 2020). Although a possible involvement of the habenula NPY-Y1 signaling in sexual and social behavior is not well known, NPY has been implicated in stress-related responses such as anxiety and depression (Shende and Desai, 2020), which are closely associated with LHb dysfunction (Hikosaka, 2010; Browne et al., 2018).

In some fish, NPY fibers or cell bodies are localized in the habenula (Chiba et al., 1996; Castro et al., 1999). While in zebrafish NPY fibers are absent in the habenula, and NPY cells located in the dorsal ENT project only to the dorsal telencephalon, but not to the habenula (Turner et al., 2016). However, single-cell RNA-seq analysis of zebrafish habenula neurons showed expression of NPY (*npy*) gene in a subpopulation of habenula (cluster 14, GABAergic neurons) and subtle expression levels of Y4 (*npy4r*) mRNA in the habenula (Pandey et al., 2018). In fish, NPY has been mainly implicated in the control of feeding behavior (Volkoff et al., 2005). However, central administration of NPY and Y4 agonist induces a reduction of locomotor activity and anxiolytic-like effect in goldfish (Matsuda et al., 2011, 2012). Further, *npy* gene-deficient zebrafish exhibit several anxiety-like behaviors, such as a decrease in social interaction and decreased locomotion in the black–white test (Shiozaki et al., 2020), which is probably similar status to passive coping (Andalman et al., 2019). Hence, NPY that is endogenously expressed within the habenula, rather than NPY derived from outside of the habenula, could be involved in modulating stress-related behaviors in teleosts.

#### Arginine Vasopressin

Arginine vasopressin (AVP) and its related peptide oxytocin are members of the evolutionarily conserved nonapeptides family derived from its ancestral vasotocin gene (Urano et al., 1992). AVP and oxytocin are well known mediators of social interaction, which influence various physiological processes and behaviors including sexual and social behaviors, circadian rhythmicity and stress response in mammals (Balment et al., 2006; Caldwell, 2017). In the brain of mammals, AVP is secreted by the magnocellular neurons of the hypothalamic supraoptic (SON) and paraventricular (PVN) nuclei, which are the main source of AVP that is released into the bloodstream (Caldwell et al., 2008). There are three major receptors for AVP: Avpr1a, Avpr1b, and Avpr2, which are distributed in specific brain regions (Caldwell et al., 2008). Avpr1a is widely distributed in several brain regions including the main SDMN components such as hippocampal formation, BNST, LS, suprachiasmatic nucleus, VTA, and hypothalamic nuclei (Ostrowski et al., 1994; Szot et al., 1994). Avpr1a (also designated as V1aR) is also expressed in the LHb (Dumais and Veenema, 2016; Rigney et al., 2020). In rodents, AVP-fibers in the habenula exhibit sexual dimorphisms with a male-dominant fiber density (De Vries et al., 1981), which are likely to originate from the BNST (De Vries and Panzica, 2006; Rood et al., 2013). In male mice, the AVP-V1aR signaling in the LHb has recently been implicated in the regulation of male-typical social communication including ultrasonic vocalization, but not other social functions such as aggression (Rigney et al., 2020). On the other hand, AVP fibers in the medial division of the LHb (LHbM) derived from the PVN evoke GABA-mediated inhibition in the LHbM, which promotes escape behavior during stress coping (Zhang et al., 2016), similar to the role of NPY on GABAergic LHb neurons (Cheon et al., 2019, 2020) (see above).

Like mammals, arginine vasotocin (AVT), the teleostean homolog of AVP, has been implicated in the modulation of a variety of social and reproductive behaviors in fish (Santangelo and Bass, 2006; Godwin and Thompson, 2012). In zebrafish, a gene expression profile study revealed that AVT expression is most closely associated with aggression, and administration of AVT inhibits dominant male and female aggression (Filby et al., 2010). In the male clownfish (*Amphiprion ocellaris*), intraperitoneal injections of V1aR antagonist significantly reduces aggression and the motivation (probability) for winning (Yaeger et al., 2014). In fish treated with V1aR antagonist, c-Fos-positive cells in the POA and the nPT are significantly reduced as compared to control fish (Yaeger et al., 2014). In fish, AVT is localized only in magnocellular and parvocellular nuclei within the POA and nPT, while AVP-fibers are distributed throughout the brain (Goodson and Bass, 2001; Parhar et al., 2001; Goodson, 2008; Godwin and Thompson, 2012). In fish, three AVP receptors: V1a1, V1a2, and V2 receptors have been identified from the Amargosa pupfish (*Cyprinodon nevadensis amargosae*) (Lema, 2010). In *A. burtoni* and a grouper, rock hind (*Epinephelus adscensionis*), V1a2 receptor (V1aR) mRNA and protein are expressed in key teleostean SDMN nodes including the habenula and the IPN (Kline et al., 2011; Huffman et al., 2012). However, the role of AVT-V1aR in the habenula and its association with the modulation of aggression in fish remains unknown.

## Role of the Habenula in Social Behaviors

The SDMN is the major neuronal network of social behaviors in vertebrates. In rodents, sex-specific and different types of social behaviors such as sexual, aggressive, and infant and parental behavior are modulated by different combinations and connectivity of nodes of the SDMN (Wei et al., 2021). Further, each node of the SDMN and its connectivity are modulated by gonadal steroid actions (Wei et al., 2021). As evident by anatomical and functional connectivities between the habenula and the SDMN and sensitivity of the habenula to sex steroids, the habenula has been implicated in the regulation of several reproductive and social behaviors in mammalian species.

### Sexual Behavior

In rodents, social behaviors including sexual behavior are triggered or suppressed by olfactory cues (*detection phase*), which involves the pathway from the olfactory inputs to the MeA (Ishii and Touhara, 2019). Next, estrogen inhibition of the MPOA contributes to dopaminergic activation of the NAc, which regulates sexual motivation and mediates the rewarding components of sexual behavior (*approaching phase*) (Micevych and Meisel, 2017). During the *investigation phase*, olfactory inputs from the vomeronasal organ (VNO) to the accessory olfactory bulb (AOB) are further transmitted to the MeA, the posterior part of the BNST and posteriormedial cortical amygdala (Spehr et al., 2006). These areas are crucial for the transition from investigation mode to consummation phase (Wei et al., 2021). Finally, the pathway from the VMHvl to the PAG is essential for the facilitation of lordosis, while a neural pathway from the ARC to the VMNvl-PAG pathway *via* the MPOA mediates transient estrogen suppression of lordosis (Tsukahara et al., 2014). As described above, the habenula has connectivities with the SDMN including the nodes of the neural circuitry of female mating behaviors, which include the MPOA, NAc, VTA, and PAG.

The habenula has been implicated in estrogenic modulation of female sexual behaviors in mammals (Pfaus et al., 1993; Lonstein et al., 2000). In female rats, the estrogen and progesterone-primed mating behavior is reduced after lesion of the habenula (Modianos et al., 1974, 1975; Rodgers and Schneider, 1979). In addition, the proceptive and receptive components of female sexual behaviors are facilitated when progesterone is implanted in the habenula (Tennent et al., 1982), while this effect is blocked when a progestin receptor antagonist is implanted into the LHb (Etgen and Barfield, 1986). In female rodents, receptive (lordosis) behavior is facilitated by progesterone following estrogen priming (Whalen, 1974; Frye and Vongher, 1999). The facilitation of reproductive behaviors are modulated by progesterone actions on the MPOA and VMHvl (Yang et al., 2013; Micevych and Meisel, 2017). In particular, the PR-positive VMHvl cells regulate female receptivity and sexual behavior and aggression in males through their projection to the PAG (Yang et al., 2013). The PAG serves as the final output structure that relays this information to hindbrain motor output neurons to induce a lordotic posture and aggression (Sakuma and Pfaff, 1979; Siegel et al., 1999; Pfaff et al., 2008). Lesions of the habenula also affects the hormonal onset of maternal behavior (Corodimas et al., 1992). On the other hand, the implantation of estrogen in the LHb does not have any effect on maternal behavior in female rats (Matthews-Felton et al., 1999), suggesting the neuromodulatory role of habenula in the onset of maternal behavior could be modulated by an estrogen-independent mechanism. Although the specific role of the habenula in the regulatory pathway of female sexual behavior remains unknown, the afferent input from the MPOA (Conrad and Pfaff, 1976) and efferent projection to the PAG (Baker and Mizumori, 2017) of LHb indicates its possible involvement in the facilitation of sexual behaviors.

In contrast to mammals, the association between reproductive/social behaviors and the habenula is limited in non-mammalian vertebrates. Nevertheless, in male newt, habenula ablation disrupts courtship behavior (Malacarne and Vellano, 1982). In frogs, there is an association between the habenula volume and seasonal reproductive activity (Kemali et al., 1990). These studies imply the involvement of the habenula in social/reproductive behaviors in non-mammalian vertebrates. In teleosts, the teleostean homolog of the SBN seems to be involved in regulating sexual behavior (Forlano and Bass, 2011). In male goldfish, a connection between the Vs and the posterior part of Vv, and the anterior part of the preoptic area (NPP), which are sensitive to gonadal steroids (Diotel et al., 2011), play important roles in sexual behavior (Koyama et al., 1984). Similarly, electric stimulation of the ventral telencephalic regions (including the Vv and Vs) and the POA (including the NPP and lateral part of the POA) elicit sexual behaviors in male and female salmon (*Oncorhynchus nerka*) (Satou et al., 1984). Similar to mammals, sexual behaviors are triggered by olfactory cues (sex pheromones) in fish (Stacey et al., 1986; Munakata and Kobayashi, 2010). In teleosts, the OB directly projects to several brain areas including the telencephalic regions (Vv and Dp) and the diencephalon (NPP and nPT) (Forlano and Bass, 2011; Kermen et al., 2013). In zebrafish, Vv neurons are broadly tuned resulting in an overlapping representation of odor categories, whereas Dp neurons respond to specific odor categories (Yaksi et al., 2009). In many fish including zebrafish, prostaglandin F_2α_ (PGF) is known to act as a female-driven reproductive hormone, which facilitates ovulation and spawning, but it also acts as a sex pheromone inducing male sexual behaviors (Sorensen et al., 1988; Pradhan and Olsson, 2015). In zebrafish, PGF acts on two olfactory receptors that are selective for PGF with different affinities in the olfactory sensory neurons, which transmit their activities to several brain regions including the Vv, NPP, the lateral hypothalamic nucleus (LH) and the caudal zone (Hc) (Yabuki et al., 2016). Further, in male fish with a gene mutation in the olfactory PGF receptor, neural activities induced by PGF is significantly reduced only in the Vv (Yabuki et al., 2016), suggesting that the Vv is primarily involved in the regulation of pheromonal regulation of sexual motivation in male fish. Although the involvement of the habenula in male sexual behaviors is not well known in fish, the habenula receives inputs from the Vv in fish (Villani et al., 1996; Yañez and Anadón, 1996; Turner et al., 2016). In addition, the habenula (dHbM) also receives direct inputs from the OB region in zebrafish (Miyasaka et al., 2009; deCarvalho et al., 2013). Hence, the habenula pathway could be potentially involved in the modulation of olfactory cues-dependent sexual behavior of fish.

### Aggressive Behavior

In fish, neurochemical, neuroendocrine, neuropharmacological, and neurogenetics aspects of aggression have been well examined (Gonçalves et al., 2017; de Abreu et al., 2019; Silva et al., 2020), which mainly involve the POA (Ogawa et al., 2021). On the other hand, aggression is strongly associated with a wide variety of social-cognitive processes such as perception, interpretation, and decision (e.g., fight or flight) (Fiske and Taylor, 1991; Allen et al., 2018). In fish, neural circuitry in social decision-making has been studied using several behavioral models (Bshary et al., 2014). Under the distinct states of social behavior by isolating the fish, having them fight a real opponent, winners only display aggressive behaviors, while losers display submissive behaviors (Ogawa et al., 2003; Teles et al., 2015, 2016). On the other hand, in the mirror-fighting model, whereby fish fight against their own image on a mirror, fish only display aggressive behaviors because they fight and cannot lose (Oliveira et al., 2005; Balzarini et al., 2014). In male *A. burtoni*, expression of neural activity marker genes such as *egr-1* and *c-fos* are increased in the SBN regions including the POA and the telencephalon in dominant fish (Burmeister et al., 2005; Maruska et al., 2013). Further, expression of *egr-1* and *c-fos* in the SBN in *A. burtoni* are most prominent in males that perceived an opportunity to ascend in social status compared to stable subordinate and dominant states (Maruska et al., 2013). In zebrafish, expression of *c-fos* and *egr-1* are induced in the telencephalic nuclei such as the Dm, Dl, Vv, and Vs and POA in social winners (winners of social of real-opponent interactions and mirror-fighters) (Teles et al., 2015). In the mudskipper, *Periophthalmus cantonensis*, *c-fos* expression is increased in the diencephalons, pons and medulla, but not in the telencephalon during aggression (Wai et al., 2006). On the other hand, in agitated fish evoked by vibratory stimuli, *c-fos* expression is increased in the Dl, Dm, thalamus, hypothalamus, pituitary and medulla (Wai et al., 2006). These results suggest that neural activity in the diencephalon and the medulla is likely to be associated with the locomotor activity during agitation and aggression, while the activities observed in the telencephalic regions could be associated with the emotional (social motivation) component. Almeida et al. (2019) revealed the rapid change in the activation pattern of the SDMN induced by the perception of outcome or fighting itself in male Mozambique tilapia (*Oreochromis mossambicus*). They found that the expression of social behavior is better explained by the overall pattern of activation of the SDMN rather than by the activity of a specific region in the SDMN (Almeida et al., 2019). Nevertheless, expression of *c-fos* in the brain revealed distinct co-activation patterns in different social environments (different social perception) (Teles et al., 2015; Almeida et al., 2019). Hence, each social perception has different sets of significant correlations among gene expression levels between different network nodes, indicative of behavior state-specific co-activation patterns.

The habenula has been implicated in aggressive behaviors in rodents and fish (Chou et al., 2016; Flanigan et al., 2017; Webster and Wozny, 2020). A functional magnetic resonance imaging in male patients with the symptom of intermittent explosive disorder (IED) shows a possible association between the habenula activity and the severity of aggressive behavior (Gan et al., 2019). However, based on animal studies, the habenula is unlikely to regulate the expression of aggressive behavior since the induction of *c-fos* in the habenula of aggressive animals was not extensive (Delville et al., 2000). On the other hand, the LHb in male mice has been recently associated with motivation and reward in the context of aggression (Golden et al., 2016; Flanigan et al., 2017). In mice, optogenetic stimulation of GABAergic LHbM neurons enhances aggression only in socially aggressive (dominant) males (Flanigan et al., 2020), indicating the inhibitory role of GABAergic LHb neurons on aggression. Importantly, these GABAergic LHb neurons coexpress receptors for estrogen, orexin and vasopressin that are implicated in aggression (Zhang L. et al., 2018). Indeed, optogenetic stimulation of orexin terminals in the LHb increases aggression, while knockdown of orexin receptor gene in GABAergic LHb neurons reduces aggression (Flanigan et al., 2020). Further, manipulation of orexin signaling within the habenula induces aggression without affecting anxiety-like behaviors (Flanigan et al., 2020). A recent study identified the involvement of dHb subnuclei in the differential regulation of social decision-making: either fight or escape in zebrafish (Chou et al., 2016). They found that silencing the dHbL reduces the likelihood of winning a fight, whereas silencing the dHbM increases the likelihood of winning (Chou et al., 2016). Interestingly, 6-day starvation in adult zebrafish facilitates winner behavior by induction of the synaptic potentiation of the dHbL-dIPN pathway which is caused by enhancement of the hypothalamic orexin signaling and the subsequent biased alternative splicing of the AMPA receptor gene in the dIPN (Nakajo et al., 2020). Although the orexin receptor (*hcrtr2*) is also present in the habenula in zebrafish (Imperatore et al., 2018), it remains unknown if orexin signaling in the habenula is involved in the conflict resolution for social dominance in zebrafish.

### Fear- and Anxiety- Like Behaviors

Fear and anxiety are defined as the response of a subject to real or potential threats that may impair its homeostasis. These responses include physiological as well as behavioral parameters (Belzung and Griebel, 2001). Clinically, anxiety is also considered a pathological state or trait (=pathological anxiety) when non-pathological ‘normal’ anxiety becomes excessive (Belzung and Griebel, 2001). Similarly, fear is an innate emotional response. Both fear and anxiety are to be quantitatively and qualitatively examined using animal models, fear- or anxiety-like behavioral phenotypes are essential. Although fear-like and anxiety-like behaviors are not social behaviors *per se*, fear and anxiety state can be closely associated with social context, which is known as social anxiety, social fear or social avoidance (Toth and Neumann, 2013). Further, social stress has been considered an excellent model to induce anxiety-like and fear-like responses (Palanza, 2001). In addition, social behavior in animal models have been widely used to study both anxiety and fear-related disorders, depending on the precise social setting employed and how the animals are manipulated (Vellucci, 1990). Hence, the social context of fear- and anxiety-like behaviors are associated with the SDMN functions. For example, amygdala hyperactivity has been observed during symptom provocation or negative emotional processing in patients with social stress-related disorders such as social anxiety, depression, specific phobia, panic disorder, and posttraumatic stress disorder (PTSD) (Amit and Tor, 2007; Godlewska et al., 2012). Nonapeptides such as AVP and oxytocin, key regulators of social functions, which play significant roles in the modulation of SDMN are implicated in the regulation of anxiety and depression (Neumann and Landgraf, 2012).

The habenula has emerged as a critical regulator of anxiety- and depression-like behaviors in animal models (Proulx et al., 2014; McLaughlin et al., 2017; Browne et al., 2018). In addition, patients diagnosed with major depressive disorder have an abnormal habenular volume (Ranft et al., 2010; Schmidt et al., 2017) and baseline activity (Sourani et al., 2012). Furthermore, a couple of clinical evidence based on deep brain stimulation in patients with treatment-resistant depression suggested the LHb as a potential therapeutic target for major depressive disorder (Sartorius et al., 2010; Wang et al., 2021). In depressive mice model induced by subchronic variable stress (SCVS) (LaPlant et al., 2009; Hodes et al., 2015), the firing rate of LHb-VTA circuit neurons is drastically increased in female mice (Zhang S. et al., 2018). In male rats, chronic stress exposure induces bilateral atrophy of the MHb and the LHb, accompanied by a reduction of the number of neurons and glial cells, and increased anxiety-like behavior (Jacinto et al., 2017). While chemical stimulation of the LHb by bilateral microinjection of kainic acid facilitates inhibitory avoidance related to anxiety, but impaired escape related to fear in rats (Pobbe and Zangrossi, 2008).

In fish, the role of habenula in anxiety- and fear-like responses has been relatively well studied using zebrafish models with established behavioral paradigms (Okamoto et al., 2012; Mathuru and Jesuthasan, 2013; Ogawa and Parhar, 2018; Bühler and Carl, 2021). For example, following chemically induced inactivation of dHb neurons (dHbl-dIPN pathway), freezing behavior is enhanced in aversive conditioning paradigms in both adult and larvae (Agetsuma et al., 2010; Lee et al., 2010). Further, disruption of the dHbl-dIPN connection but not the dHbm-vIPN pathway leads to anxiety-like behavior, suggesting that the dHbl neurons are sensitive to aversive stimuli (Duboué et al., 2017). The dHbl-dIPN pathway has also been implicated in social decision-making, but it is related to social conflict resolution (winner or loser) (Chou et al., 2016). On the other hand, recent studies revealed that the vHb appears to play an essential role in social decision-making related to fear and anxiety in zebrafish (Okamoto et al., 2021). In zebrafish chronically under threat, vHb neurons are tonically activated in the presence of aversive cues, which is accompanied by activated serotonergic raphe neurons (Amo et al., 2014). This neuronal activity of vHb-serotonin pathway is suggested to represent the expectation of a social threat (social risk evaluation). Furthermore, the place avoidance behavior can be induced by optogenetic stimulation of vHb neurons (Amo et al., 2014). Hence, the vHb-MR circuit is essential for representing expected danger and behavioral programming, allowing adaptively avoiding potential social hazards (Amo et al., 2014).

As described above (see section “Kisspeptin”), kisspeptin (Kiss1) and its receptor (Kiss1R) are exclusively expressed in the zebrafish vHb (Kitahashi et al., 2009). Central administration of Kiss1 (zebrafish Kiss1-15 peptides) increases the expression of genes associated with serotonin (Ogawa et al., 2012), which indicates Kiss1 could indirectly act on serotonergic neurons residing the vaMR. Further, in zebrafish administered with Kiss1, odorant cue (alarm substances) induced fear responses including erratic movement followed by freezing behavior is significantly reduced (Ogawa et al., 2014). A similar effect is observed when the Kiss1 neurons are chemically ablated (Ogawa et al., 2014) or *Kiss1* gene is mutated (Sivalingam, 2019). Interestingly, Kiss1 gene mutant zebrafish exhibits avoidance learning impairment (Lupton et al., 2017). Similarly, according to Okamoto et al. (2021), the vHb-silenced fish impair social avoidance learning. Under a social conflict situation, the socially subordinate fish tends to have less proximity to the socially dominant individual to avoid being attacked by the dominant (= social avoidance learning) (Krypotos et al., 2015). However, the vHb-silenced fish repeatedly swims back to the center of the tank where the dominant (winner) fish is swimming, and continues to receive attacks by the dominant fish (Okamoto et al., 2021). These observations suggest the possible role of Kiss1-Kiss1R signaling in the vHb in the modulation of stress coping in zebrafish. Although the mechanism underlying the neuromodulatory role of Kiss1 remains unclear, an electrophysiological characterization in larval zebrafish has shown concentration-dependent dual (stimulatory and inhibitory) effects of Kiss1 peptides on vHb neural activity: vHb neurons are depolarized at low concentrations (10∼100 nM), whereas they are hyperpolarized at high concentrations (1∼5 μM) (Lupton et al., 2017). In larval zebrafish, activation of vHb neurons suppresses downstream serotonergic neurons and induces passive coping (reduction of mobility under stress), whereas inhibition of these neurons prevents passivity (Andalman et al., 2019). We have previously shown that exposure to cold stress (15°C) significantly induces *Kiss1* gene expression in the zebrafish habenula (Shahjahan et al., 2013). Hence, habenula Kiss1 could act as a sensor for stressful or socially aversive conditions and modulate the subsequent stress-coping behavior such as mobility (avoidance), anxiety and aversion-associated learning.

Apart from Kiss1 signaling, the habenula expresses a number of molecules including neurotransmitters and their transporters and receptors, neuropeptides and neuropeptide receptors, and channels, which were recently revealed by several habenula-specific single-cell RNA-seq analyses in mammals (Hashikawa et al., 2020; Levinstein et al., 2020; Wallace et al., 2020) and fish (Pandey et al., 2018). However, their molecular and functional roles within the vHb remain unelucidated. We have recently identified the expression of GPR139 in the vHb of zebrafish and found that activation of GPR139 in the vHb could be involved in the modulation of fear learning and decision-making process (Roy et al., 2021). As treatment with a human GPR139-selective agonist had no effect on odorant cue-induced fear response, GPR139 and Kiss1 signaling in the vHb are independently involved in aversion responses (Roy et al., 2021).

## Summary

Social and sexual behaviors are collectively controlled by the specialized SDMN circuitry in vertebrates. SDMN is structurally, biochemically and possibly functionally conserved from non-mammalian vertebrates to humans. The SDMN circuitry has numerous connectivity outside of the SDMN. Recently, the habenula, an evolutionarily highly conserved epithalamic structure, has emerged as a possible key brain region in decision-making behaviors in a social interaction context. Accumulated neuroanatomical and neurochemical evidence suggests that there is a functional linkage between the habenula and the SDMN in mammals and fish. For example, there is reciprocal connectivity between the habenula and components of the SDMN. The habenula receives neuronal inputs from several neuropeptides regulating social and reproductive behaviors. The connectivity between the habenula and the Vv could be potentially involved in the modulation of olfactory cues-dependent sexual motivation in fish. Recent behavioral and neurogenetic studies in zebrafish have revealed that different habenular subnuclei selectively regulate the different states of social behaviors. The dHb-dIPN pathway modulates experience-dependent behavioral response (anxiety) toward social threat. On the other hand, the vHb-serotonergic pathway is involved in adaptive learning of social avoidance. Taken together, the habenula plays a crucial role in controlling different states of social behaviors and hence, it may be considered an indispensable node of the SDMN in vertebrates.

## Author Contributions

SO: conceptualization, writing – original draft preparation, and illustration. ISP: writing – review and editing. Both authors: contributed to the article and approved the submitted version.

## Conflict of Interest

The authors declare that the research was conducted in the absence of any commercial or financial relationships that could be construed as a potential conflict of interest.

## Publisher’s Note

All claims expressed in this article are solely those of the authors and do not necessarily represent those of their affiliated organizations, or those of the publisher, the editors and the reviewers. Any product that may be evaluated in this article, or claim that may be made by its manufacturer, is not guaranteed or endorsed by the publisher.
